# Supplementary material for: Neoadjuvant Concurrent Chemoradiotherapy Versus Neoadjuvant Chemotherapy in Thymic Epithelial Tumors: A Propensity Score-Matched Analysis
Source: Cancers (Basel). 2025 Dec 27;18(1):85. doi: 10.3390/cancers18010085 (PMC12785132; doi:10.3390/cancers18010085)
Supplement: Supplementary file 1 [file cancers-18-00085-s001.zip › cancers-4045351-supplementary.pdf]

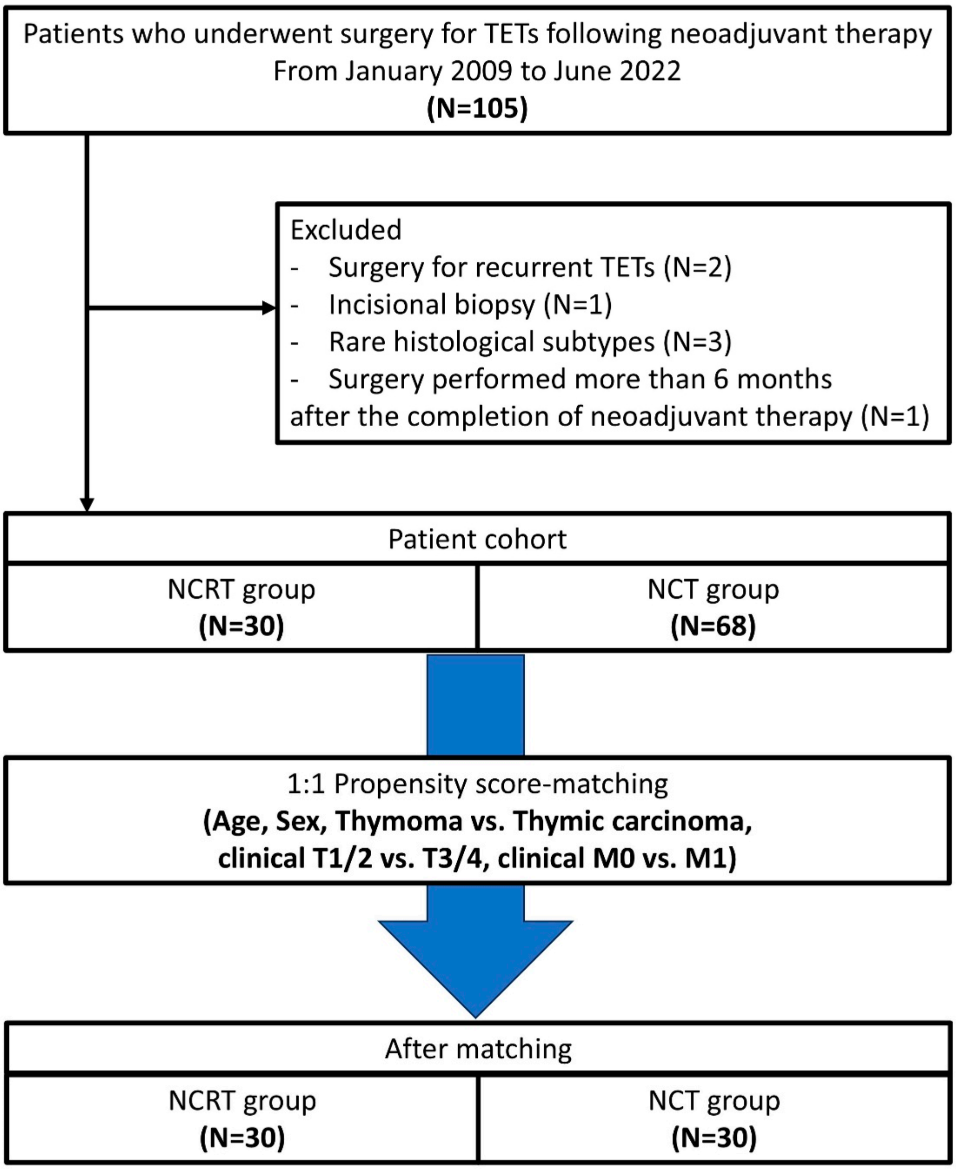

Figure S1. Consort diagram of the study population.

NCRT : neoadjuvant chemoradiotherapy, NCT : neoadjuvant chemotherapy, TET : thymic epithelial tumor

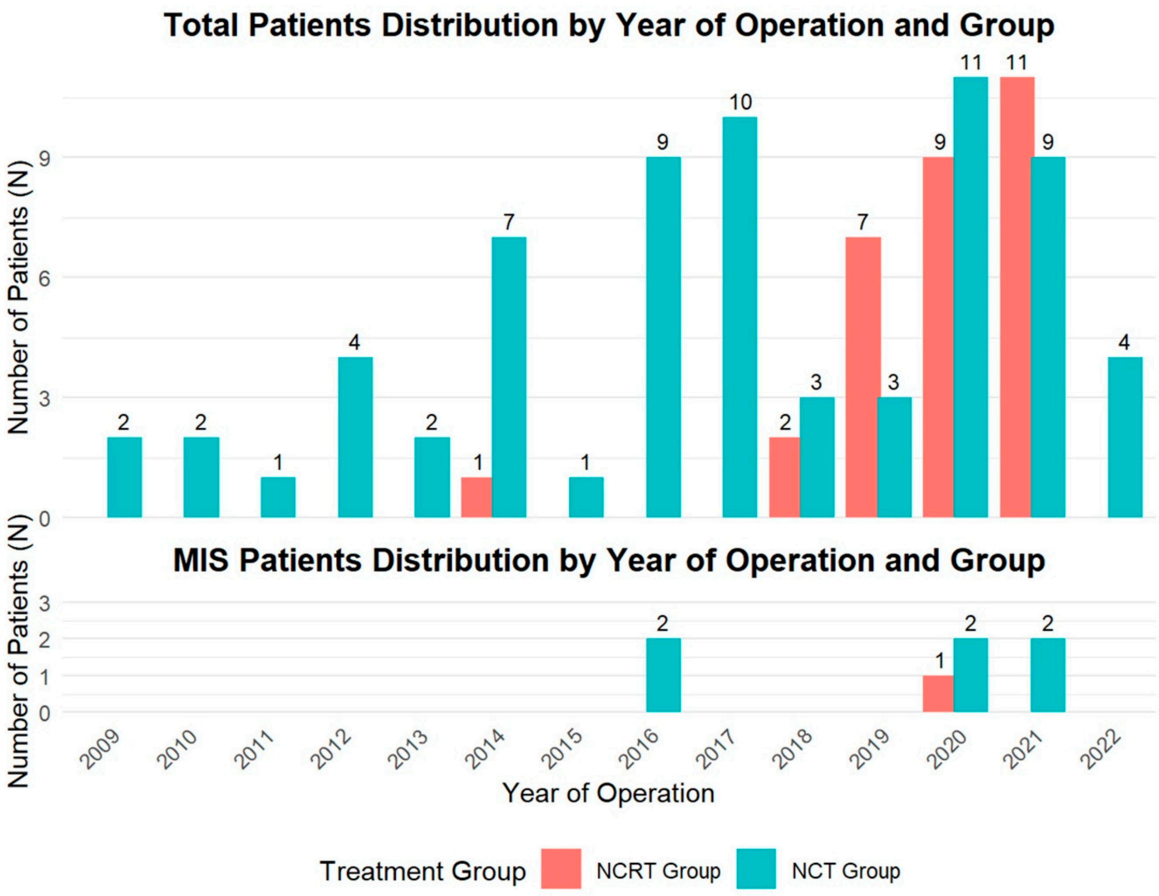

Figure S2. Patients distribution by year of operation and group.

MIS : minimally invasive surgery, NCRT : neoadjuvant chemoradiotherapy, NCT : neoadjuvant chemotherapy

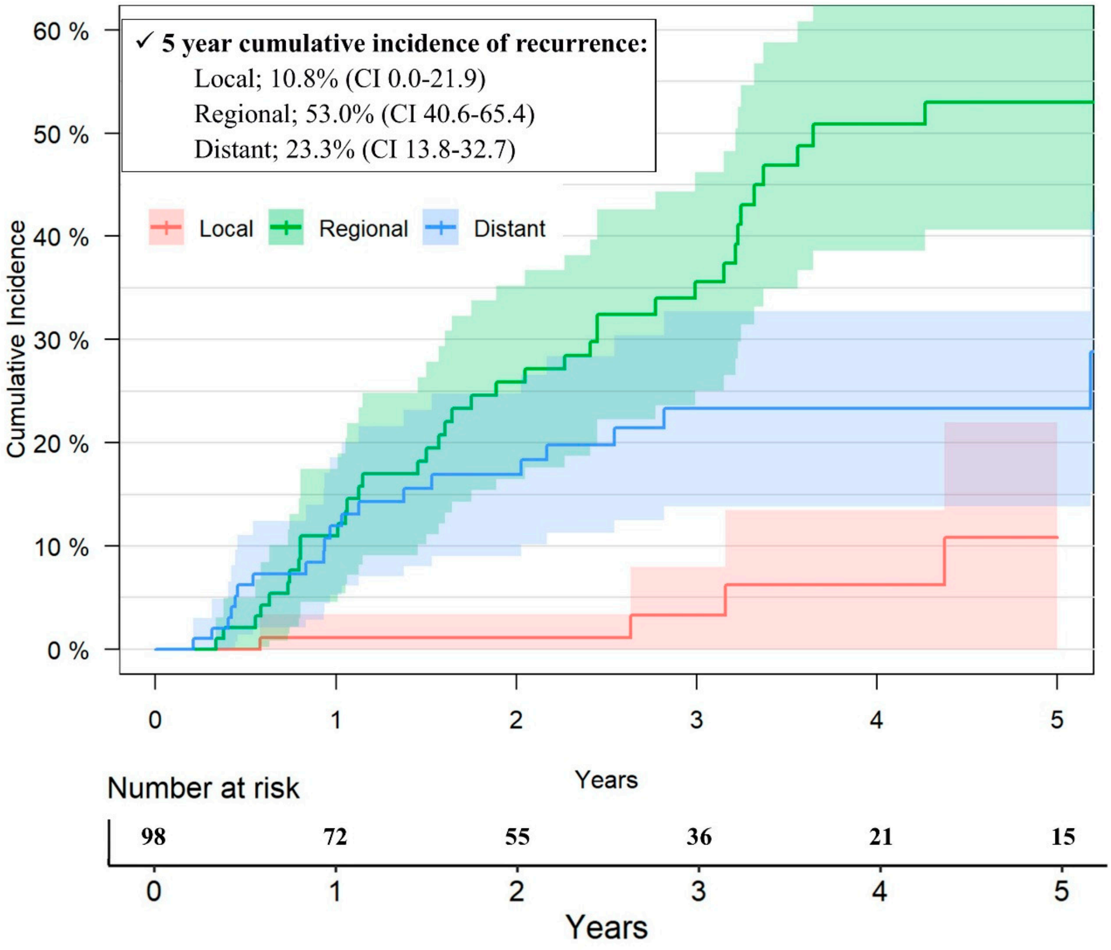

Figure S3. Cumulative incidence rate curves for local, regional, and distant recurrence patterns with death regarded as a competing risk in pre-matched population.

CI : confidence interval (95%)

Table S1. Matching variables.

	Pre-matched patients			PS-matched patients		
	NCRT (n = 30)	NCT (n = 68)	SMD	NCRT (n = 30)	NCT (n = 30)	SMD
Age	53.5 ± 9.7	54.1 ± 12.7	-0.063	53.5 ± 9.7	53.3 ± 11.6	-0.055
Male (vs. Female)	20 (66.7%)	41 (60.3%)	0.135	20 (66.7%)	17 (56.7%)	0.141
Thymic carcinoma (vs. Thymoma)	20 (66.7%)	22 (32.4%)	**0.728**	20 (66.7%)	18 (60.0%)	0.141
Clinical T3/4 (vs. T1/2)	27 (90.0%)	50 (73.5%)	**0.540**	27 (90.0%)	26 (86.7%)	0.110
Clinical M1 (vs. M0)	7 (23.3%)	45 (66.2%)	**-1.013**	7 (23.3%)	10 (33.3%)	-0.158

Values with an absolute SMD of 0.2 or higher are highlighted in bold.

Table S2. Detailed surgical procedures.

Variable	Pre-matched patients				PS-matched patients		
	Total (n=98)	NCRT group (n=30)	NCT group (n=68)	P value	NCRT group (n=30)	NCT group (n=30)	P value
**Intraop. CPB/ECMO support**	26.5% (n=26)	46.7% (n=14)	17.6% (n=12)	0.003	46.7% (n=14)	16.7% (n=5)	0.012
**Surgical approach**				0.200			0.610
S, S+V, S+N	78.6% (n=77)	93.3% (n=28)	72.1% (n=49)		93.3% (n=28)	90.0% (n=26)	
S+T	7.1% (n=7)	0% (n=0)	10.3% (n=7)		0% (n=0)	3.3% (n=2)	
T	6.1% (n=6)	3.3% (n=1)	7.4% (n=5)		3.3% (n=1)	0% (n=0)	
C	1.0% (n=1)	0% (n=0)	1.5% (n=1)		0% (n=0)	3.3% (n=1)	
MIS	7.1% (n=7)	3.3% (n=1)	8.8% (n=6)		3.3% (n=1)	3.3% (n=1)	
**Combined resection**							

Aorta	7.1% (n=7)	13.3% (n=4)	4.4% (n=3)	0.196	13.3% (n=4)	6.7% (n=2)	0.671
- R1 at aorta	14.3% (n=1)	0% (n=0)	33.3% (n=1)	0.429	0% (n=0)	50.0% (n=1)	0.333
PA	3.1% (n=3)	6.7% (n=2)	1.5% (n=1)	0.221	6.7% (n=2)	0% (n=0)	0.492
SVC	32.7% (n=32)	40.0% (n=12)	29.4% (n=20)	0.303	40.0% (n=12)	40.0% (n=12)	1.000
Innominate vein	43.9% (n=43)	60.0% (n=18)	36.8% (n=25)	0.033	60.0% (n=18)	40.0% (n=12)	0.121
- R1 at innominate vein	4.7% (n=2)	11.1% (n=2)	0% (n=0)	0.169	11.1% (n=2)	0% (n=0)	0.503
Arch vessel	2.0% (n=2)	3.3% (n=1)	1.5% (n=1)	0.521	3.3% (n=1)	0% (n=0)	1.000
Pericardium	65.3% (n=64)	73.3% (n=22)	61.8% (n=42)	0.267	73.3% (n=22)	50.0% (n=15)	0.063
- R1 at pericardium	4.7% (n=3)	4.5% (n=1)	4.8% (n=2)	0.344	4.5% (n=1)	6.7% (n=1)	1.000
Lung	81.6% (n=80)	70.0% (n=21)	86.8% (n=59)	0.048	70.0% (n=21)	86.7% (n=26)	0.117
Phrenic nerve	34.7%	53.3%	26.5%	0.010	53.3%	26.7%	0.035

	(n=34)	(n=16)	(n=18)		(n=16)	(n=8)	
Chest wall	10.2% (n=10)	10.0% (n=3)	10.3% (n=7)	1.000	10.0% (n=3)	13.3% (n=4)	1.000
- R1 at chest wall	30.0% (n=3)	0% (n=0)	42.9% (n=3)	0.475	0% (n=0)	50.0% (n=2)	0.429
Vagus nerve	7.1% (n=7)	10.0% (n=3)	5.9% (n=4)	0.672	10.0% (n=3)	10.0% (n=3)	1.000
Diaphragm	16.3% (n=16)	0% (n=0)	23.5% (n=16)	0.002	0% (n=0)	10.0% (n=3)	0.237
Epicardium	2.0% (n=2)	0% (n=0)	2.9% (n=2)	1.000	0% (n=0)	0% (n=0)	1.000

C; clamshell incision, CPB; cardiopulmonary bypass, ECMO; extracorporeal membrane oxygenation, MIS; minimal invasive surgery including VATS or Robot, N; neck incision, NCRT; neoadjuvant chemoradiotherapy, NCT; neoadjuvant chemotherapy, PS; propensity score, S; sternotomy, SVC; superior vena cava, T; thoracotomy, V; VATS

Table S3. Postoperative complications.

Variable	Pre-matched patients				PS-matched patients		
	Total (n=98)	NCRT group (n=30)	NCT group (n=68)	P value	NCRT group (n=30)	NCT group (n=30)	P value
**Cx**	46.9% (n=46)	40.0% (n=12)	50.0% (n=34)	0.361	40.0% (n=12)	50.0% (n=15)	0.436
**Cx Gr≥3**	25.5% (n=25)	23.3% (n=7)	26.5% (n=18)	0.743	23.3% (n=7)	26.7% (n=8)	1.000
**Vocal cord palsy**	15.3% (n=15)	13.3% (n=4)	16.2% (n=11)	1.000	13.3% (n=4)	16.7% (n=5)	1.000
**Pneumonia**	9.2% (n=9)	10.0% (n=3)	8.8% (n=6)	1.000	10.0% (n=3)	6.7% (n=2)	1.000
**Bleeding**	3.1% (n=3)	0% (n=0)	4.4% (n=3)	0.551	0% (n=0)	3.3% (n=1)	1.000
**Thromboembolism**	2.0% (n=2)	3.3% (n=1)	1.5% (n=1)	0.521	3.3% (n=1)	3.3% (n=1)	1.000
**Stroke**	1.0% (n=1)	3.3% (n=1)	0% (n=0)	1.000	3.3% (n=1)	0% (n=0)	1.000
**Chylo-thorax**	8.2% (n=8)	13.3% (n=4)	5.9% (n=4)	0.244	13.3% (n=4)	6.7% (n=2)	0.671
**Pleural effusion**	2.0% (n=2)	0% (n=0)	2.9% (n=2)	1.000	0% (n=0)	3.3% (n=1)	1.000

**Pericardial effusion**	3.1% (n=3)	3.3% (n=1)	2.9% (n=2)	1.000	3.3% (n=1)	3.3% (n=1)	1.000
**Arrhythmia**	11.2% (n=11)	6.7% (n=2)	13.2% (n=9)	0.495	6.7% (n=2)	10.0% (n=3)	1.000
**Wound problem**	3.1% (n=3)	3.3% (n=1)	2.9% (n=2)	1.000	3.3% (n=1)	3.3% (n=1)	1.000

Cx; complication, Gr; grade, NCRT; neoadjuvant chemoradiotherapy, NCT; neoadjuvant chemotherapy, PS; propensity score

Table S4. Postoperative complications by the use of intraoperative CPB/ECMO support.

Variable	Pre-matched patients		
	CPB/ECMO (-) (n=72)	CPB/ECMO (+) (n=26)	P value
**Cx**	44.4% (n=32)	53.8% (n=14)	0.410
**Cx Gr≥3**	25.0% (n=18)	26.9% (n=7)	0.847
**Vocal cord palsy**	12.5% (n=9)	23.1% (n=6)	0.215
**Pneumonia**	6.9% (n=5)	15.4% (n=4)	0.240
**Bleeding**	4.2% (n=3)	0% (n=0)	0.563
**Thromboembolism**	0% (n=0)	7.7% (n=2)	0.068
**Stroke**	1.4% (n=1)	0% (n=0)	1.000
**Chylothorax**	5.6% (n=4)	15.4% (n=4)	0.203
**Pleural effusion**	2.8% (n=2)	0% (n=0)	1.000
**Pericardial effusion**	1.4% (n=1)	7.7% (n=2)	0.171
**Arrhythmia**	8.3% (n=6)	19.2% (n=5)	0.154
**Wound problem**	2.8% (n=2)	3.8% (n=1)	1.000

Table S5. Univariable and multivariable analysis for the risk factors of overall survival in pre-matched patients using Cox regression model.

Risk factors	Univariable analysis			Multivariable analysis		
	HR	95% CI	p-value	HR	95% CI	p-value
**Age**	1.030	0.994 – 1.068	0.105			
**Male**	2.720	1.019 – 7.258	0.046			
**Ever smoker**	2.346	1.053 – 5.227	0.037	2.843	1.214 – 6.657	0.016
**Myasthenia gravis**	2.072	0.703 – 6.109	0.187	3.847	1.014 – 14.590	0.048
**Thymic carcinoma**	2.417	1.079 – 5.418	0.032	3.656	1.406 – 9.509	0.008
**Mass size in CT (mm)**	0.997	0.980 – 1.013	0.682			
**Clinical stage**						
- **IV vs. II+III**	1.694	0.676 – 4.248	0.261			
**NCRT**	0.857	0.312 – 2.350	0.764			
**Pathologic stage**			0.068			
- **III vs. I+II**	0.341	0.062 – 1.873	0.216			
- **IV vs. I+II**	1.754	0.591 – 5.208	0.312			
- **IV vs. III**	5.155	1.196 –22.222	0.028			
**Complete resection**	0.306	0.136 – 0.688	0.004	0.301	0.130 – 0.700	0.005
**TRG**	0.835	0.472 – 1.479	0.537			
**Necrosis (%)**	0.989	0.963 – 1.016	0.436			

**WHO cell type (thymoma)**			
- **B3 vs. B2, B1, AB, A**	0.559	0.141 – 2.216	0.408
**Adjuvant CTx**	1.456	0.540 – 3.923	0.458
**Adjuvant RTx**	1.235	0.551 – 2.766	0.608
**Cx Gr≥3**	2.578	1.153 – 5.766	0.021

Table S6. Univariable and multivariable analysis for the risk factors of recurrence-free survival in pre-matched patients using Cox regression model.

Risk factors	Univariable analysis			Multivariable analysis		
	HR	95% CI	p-value	HR	95% CI	p-value
**Age**	1.006	0.984 – 1.029	0.608			
**Male**	1.496	0.912 – 2.454	0.111			
**Ever smoker**	1.907	1.149 – 3.167	0.013	2.075	1.235 – 3.486	0.006
**Myasthenia gravis**	1.198	0.547 – 2.621	0.651			
**Thymic carcinoma**	1.243	0.776 – 1.990	0.365			
**Mass size in CT (mm)**	1.003	0.995 – 1.011	0.538			
**Clinical stage**						
- **IV vs. II+III**	1.804	1.064 – 3.057	0.028			
**NCRT**	0.932	0.547 – 1.587	0.794			
**Pathologic stage**			0.003			0.009
- **III vs. I+II**	0.934	0.428 – 2.037	0.864	1.007	0.454 – 2.232	0.986
- **IV vs. I+II**	2.253	1.162 – 4.369	0.016	2.179	1.087 – 4.369	0.028
- **IV vs. III**	2.410	1.337 – 4.348	0.003	2.165	1.195 – 3.922	0.011
**Complete resection**	0.427	0.248 – 0.734	0.002	0.528	0.301 – 0.927	0.026
**TRG**	0.851	0.609 – 1.190	0.346			
**Necrosis (%)**	0.998	0.985 – 1.012	0.811			

**WHO cell type (thymoma)**			
- **B3 vs. B2, B1, AB, A**	1.206	0.646 – 2.252	0.556
**Adjuvant CTx**	1.194	0.648 – 2.197	0.570
**Adjuvant RTx**	1.105	0.674 – 1.812	0.692
**Cx Gr≥3**	1.515	0.893 – 2.570	0.123
